# Key hepatic metabolic pathways are altered in germ-free mice during pregnancy

**DOI:** 10.1371/journal.pone.0248351

**Published:** 2021-03-12

**Authors:** Lyrialle W. Han, Yuanyuan Shi, Alison Paquette, Lu Wang, Theo K. Bammler, Qingcheng Mao

**Affiliations:** 1 Department of Pharmaceutics, School of Pharmacy, University of Washington, Seattle, Washington, United States of America; 2 Department of Medicinal Chemistry, School of Pharmacy, University of Washington, Seattle, Washington, United States of America; 3 Center for Developmental Biology and Regenerative Medicine, Seattle Children’s Research Institute, and Department of Pediatrics, University of Washington, Seattle, Washington, United States of America; 4 Department of Environmental and Occupational Health Sciences, School of Public Health, University of Washington, Seattle, Washington, United States of America; University of Iowa, UNITED STATES

## Abstract

Pregnancy is associated with metabolic changes to accommodate the mother and her growing fetus. The microbiome has been shown to modulate host metabolism of endogenous and exogenous substances. However, the combined effects of pregnancy and the microbiome on host metabolism have not been investigated. The objective of this study was to investigate how the microbiome affects overall hepatic metabolic processes during pregnancy. We assessed these changes within 4 groups of C57BL/6 mice: conventional non-pregnant, conventional pregnant, germ-free non-pregnant, and germ-free pregnant mice. We performed RNA-seq analysis on liver tissues and LC-MS/MS analysis of the plasma to assess the effects of pregnancy and the microbiome on hepatic transcriptome and untargeted plasma metabolome to describe metabolic changes as results of both pregnancy and lack of microbiome. By integrating transcriptomics and metabolomics data, we identified eight metabolic pathways that were significantly enriched for differentially expressed genes associated with pregnancy in both conventional and germ-free mice. Notably, of the eight pathways, 4 pathways (retinol metabolism, arachidonic acid metabolism, linoleic acid metabolism, and steroid hormone biosynthesis) which are all critical for normal pregnancy and fetal development were affected by the germ-free status in pregnant mice, but not at all in non-pregnant mice, indicating that the alterations in these four pathways caused by the lack of microbiome are unique for pregnancy. These results provide novel insight into the role of the microbiome in modulating host metabolic processes critical for maternal health and fetal development during pregnancy.

## Introduction

Pregnancy is a physiological process with numerous changes in the maternal body to accommodate the developing fetus. Maternal metabolic processes adapt to the growth of the fetus and its expanding needs. Throughout gestation, the maternal body has altered levels of lipoproteins, cholesterol, triglycerides, and phospholipids to meet the growing fetus’s nutritional demands [1]. These metabolic changes in turn trigger the immune system to react to the pregnancy by increasing proinflammatory cytokine levels to further enhance energy storage [1, 2]. Another major change that comes with pregnancy is a shift in microbiome composition. For example, studies have shown that the gut microbiome composition in women during pregnancy is associated with an increase in Proteobacteria and Actinobacteria, relative to other species, from the first trimester through the third trimester [3]. The richness of the gut microbiome composition is found to be reduced in late pregnancy, with a pronounced increase in bacteria associated with inflammatory processes [2–4].

The microbiome has become an important area of research in recent years because of increasing evidence of the capability of bacteria to modulate host metabolic processes via various microbiota-metabolic axes. For example, gut bacteria play a critical role in the enterohepatic circulation of endogenous compounds, such as short-chain fatty acids and primary bile acids, which are crucial for host health [5, 6]. The gut microbiome has also been shown to modulate host xenobiotic metabolism both by directly metabolizing compounds in the intestine or indirectly via the production of metabolites that interact with nuclear receptors to regulate the expression of host xenobiotic metabolizing genes [5, 6].

Over the last decade, gut dysbiosis (imbalance or disruption of the gut microbiome) has been observed to be on the rise in the westernized populations, possibly due to changes in diet and a more sedentary lifestyle [5, 7, 8]. This adds another layer of variability to the host-gut supraorganism interactions and consequent effects on host wellbeing. Given the gut microbiome composition shifts dynamically as gestation progresses, the impact of such changes in the microbiome on host metabolic processes during pregnancy should also be explored [2, 9–11].

We have previously used germ-free mice to investigate how the microbiome affects hepatic drug processing genes during pregnancy and found that the lack of microbiome can have a significant impact on the expression and/or activity of key hepatic drug processing genes during pregnancy [12]. For example, mouse hepatic cytochrome P450 (CYP) *Cyp3a* genes have multiple isoforms (*Cyp3a11*, *Cyp3a16*, *Cyp3a41*, *Cyp3a44)*, and *Cyp3a11* is considered the murine ortholog of human *CYP3A4*, a major human CYP enzyme known to metabolize numerous endogenous and exogenous substrates. We found that the overall CYP3A activity was significantly induced by pregnancy in both conventional (CV) and germ-free (GF) mice; however, the magnitude of induction was drastically decreased several-fold in GF mice compared to CV mice. This could lead to altered pharmacokinetics and pharmacodynamics of drugs that are metabolized by CYP3A enzymes in pregnant women by altering the microbiome composition or due to dysbiosis during pregnancy should the same effects of the microbiome occur in humans. The impact of the microbiome and its host fitness interactions are not limited to hepatic metabolism of drugs and xenobiotics. In fact, previous studies have demonstrated that the gut microbiome can modulate overall host metabolic processes as well [5]. Abnormal metabolic changes that are not natural to the progression of pregnancy can pose high risks to both the mother and the fetus, such as increased risk for gestational hypertension, gestational diabetes, or neurodevelopmental disorders later in life [2, 13]. At present, very little information is available on what metabolic pathways important for pregnancy and fetal growth are influenced by the microbiome. By gaining insights into these changes, we may better understand the sources of inter-individual variability of pregnancy-related diseases and therapeutic effects of medications during pregnancy. In the previous study, we used targeted transcriptomic, proteomic, and metabolomic approaches to determine the effects of the microbiome on the expression of hepatic drug processing genes during pregnancy [12]. However, the effects of pregnancy and the microbiome on overall hepatic metabolism have yet to be determined. Thus, the objective of this study was to explore the influence of the microbiome on overall maternal hepatic metabolic pathways during pregnancy using CV and GF mice. We analyzed the changes in overall hepatic gene expression and maternal plasma metabolites using RNA-seq transcriptomics and LC-MS/MS-based untargeted metabolomics individually. We then integrated transcriptomics and metabolomics data for a joint pathway analysis to identify hepatic metabolic pathways that are uniquely altered by the microbiome during pregnancy.

## Materials and methods

### Mice and animal studies

Four groups of C57BL/6 mice were used: conventional non-pregnant (CVNP) mice, conventional pregnant (CVP) mice, germ-free non-pregnant (GFNP) mice, and germ-free pregnant (GFP) mice. Conventional (CV) C57BL/6J mice were purchased from The Jackson Laboratory (JAX stock #000664). Germ-free (GF) C57BL/6 mice were descendants of the original colony from the National Gnotobiotic Rodent Resource Center of the University of North Carolina at Chapel Hill which was derived from the Jackson Labs C57BL/6J embryos. Animal care and use were all in accordance with the Guide for the Care and Use of Laboratory Animals published by National Research Council. This animal protocol was approved by the Institutional Animal Care and Use Committee of University of Washington (protocol #4035–04).

Details of the animal studies were the same as previously described [12]. Briefly, all animals (pregnant and non-pregnant mice) were maintained with the same autoclaved diet, non-acidified water, and autoclaved bedding. Food and water were provided to all mice *ad libitum*. Age matched CV mice were mated overnight at 8 weeks of age. In the morning after overnight mating, male mice were separated from female mice. The day on which male and female mice were put together for mating was considered gestation day 0 (gd 0). We noted that the breeding ability of GF mice of this C57BL/6 mouse strain was much lower than that of CV C57BL/6J mice. Therefore, due to difficulties achieving pregnancy with overnight mating technique with GF mice, GF female mice were mated for 72 h with GF male mice and the second day was considered gd 0. All plasma samples and liver tissues were collected from non-pregnant female mice and pregnant mice on gd 15 (or at equivalent times for non-pregnant mice) as previously described [12]. Liver tissues and plasma samples were frozen immediately in liquid N_2_ and kept at -80°C until further analysis.

### RNA-seq transcriptomics analysis

Total RNA was extracted from frozen liver tissues from CV and GF mice (n = 6, 5, 6, and 5 for CVNP, CVP, GFNP, and GFP mice, respectively) and sequenced as previously described [12]. Briefly, we performed paired-end RNA sequencing using Illumina NovaSeq 6000 and prepared the transcriptomic library using NEBNext^®^ Ultra^™^ RNA Library Prep Kit for Illumina^®^. The reads were aligned to mouse GRCm38.p6 transcriptome and summarized using the Bioconductor tximport package in R (v1.10.1). Then, data was filtered for consistently low basal expression genes using edgeR (v3.24.3). After this filtering step, a total of 18,849 genes remained. Differentially expressed genes were identified by fitting a quasi-likelihood negative binomial generalized log-linear model [14, 15], followed by quasi-likelihood F tests for each comparison (CVP vs. CVNP; GFP vs. GFNP; GFNP vs. CVNP; GFP vs. CVP). We have previously published the complete method of RNA-seq data analysis [12]. A false discovery rate (FDR) of 0.1 and minimum fold-change of 2 were used to identify differentially expressed genes (DEGs). Raw RNA-seq data used in this study were deposited in the National Center for Biotechnology Information Gene Expression Omnibus data repository under accession number GSE143391.

### Untargeted metabolomics analysis

Frozen plasma samples from CV and GF mice (n = 6, 6, 6, and 5 for CVNP, CVP, GFNP, and GFP mice, respectively) were used to perform untargeted metabolomics analysis. Plasma metabolite extraction was identical to plasma steroid hormone extraction as we previously described [12]. The metabolomics analysis was conducted on UPLC-MS/MS (SCIEX Triple Quadrupole 5600 system (Framingham, WA) coupled to an ACQUITY UPLC system (Waters Technologies, Milford, MA). Samples were injected onto the column (ACQUITY UPLC HSS T3 1.8μm, C18 100A; 100x2.1 mm, Waters, Milford, MA). The mobile phase was consisted of 0.1% Formic acid in water (A)-0.1% formic acid in acetonitrile (B) and running with 0.3 ml/min flow rate. Gradient program was as follows: mobile phase B 5%-36% (0–5 min), 36–95% (5–20 min), 95% (20–22 min) and back to 5% for 2min equilibration. MS acquisition was achieved by using the following set of parameters: source temperature, 400°C, curtain gas flow, 30 and the two ion source gas flows were set at 40 (arbitrary unit). The MS spectra were acquired in the mass range of 100–1,500 m/z and fragments were acquired in the mass range of 50–1,500 m/z. Then, the data were imported to the Progenesis QI software (Waters Corporation) for data processing. During the procedure, the software carried out deconvolution, alignment, peak picking, and statistical analysis, identification, and compound measurement with corresponding intensities for all the detected peaks from each data file in the dataset [16]. The peak picking conditions were set as follows: all runs, limits (automatic), sensitivity (3), chromatographic peak width (minimum peak width), and retention time (0.5 to 22.0 min). A total of 4936 compounds from positive mode and 5505 compounds from negative mode were initially selected within this retention time period. Different adduct ion forms were applied to deconvolute the spectral data. Metabolites significantly associated with each group of mice were identified using one-way analysis of variance (ANOVA) with significance defined as a threshold of *p* < 0.1 and FDR < 0.1 and following EZinfo 3.0, which allows preliminarily screening of potential biomarkers and identifying group differences via orthogonal partial least square-discriminant analysis (OPLS-DA) and principal component analysis (PCA). The parameters, R^2^Y and Q^2^ (>0.85), were used to evaluate the quality of the model. Candidate compounds of significance were filtered under two conditions, that is, VIP values (VIP > 1) and max fold change ≥ 2. The potential metabolites were reprinted on the Progenesis QI software and created tags. Significant variables were identified and confirmed by comparing MS data, MS/MS fragments and elemental compositions (H (0−50), C (0−50), N (0− 5), and O (0−30), precursor tolerance 10 ppm, and isotope similarity 95%) with the biochemical databases, HMDB (http://www.hmdb.ca/) with both precursor tolerance and fragment tolerance 10 ppm to identify and confirm candidate metabolites. A threshold of 0.1 FDR was applied to filter out false-positives, and a minimum fold change of 2 was also applied to identify differentially produced metabolites between groups.

### Joint pathway analysis

MetaboAnalyst 4.0 (http://metaboanalyst.ca) was used to perform joint pathway analysis [17, 18]. Differentially expressed genes (DEGs) (imported as official gene symbol) and differentially produced metabolites (imported as HMDB ID) between CVP, CVNP, GFP, and GFNP mice were used as integrated input for the analysis. Inclusion criteria for genes and metabolites were FDR of 0.1 and a minimum 2-fold change in at least one group comparison. We used metabolic pathways in Kyoto Encyclopedia of Genes and Genomes (KEGG) database (Version Oct2019) for Mus musculus. A total of 1182 (out of 1231) genes and 1602 (out of 2277) metabolites for the CVP versus CVNP group, 797 (out of 859) genes and 1580 (out of 2223) metabolites for the GFP versus GFNP group, 20 (out of 20) genes and 1602 (out of 2277) metabolites for the GFNP versus CVNP group, and 18 (out of 18) genes and 2469 (out of 3367) metabolites for the GFP versus CVP group were successfully mapped to the KEGG database and used for subsequent pathway enrichment analysis. Fisher’s exact tests and degree centrality [17, 18] were used to determine pathway enrichment and reported with pathway-level weighted FDR-adjusted *p*-value. All pathways with FDR < 0.1 were considered significant. Impact score was calculated based on degree centrality algorithms. The pathway impact score reflects the cumulative percentage of the degree centrality of each differentially expressed metabolite and/or gene within the network. Degree centrality is a measure of the number of links between each node; and in this context the node represents a gene or metabolite. Those keg compounds that are central to the pathway and have more connections would thus have a higher degree centrality measure. Thus, pathways with higher impact scores had more centrally important genes or metabolites associated with each phenotype.

## Results

### Changes in hepatic gene expression in CV and GF mice by pregnancy

**Fig 1:**
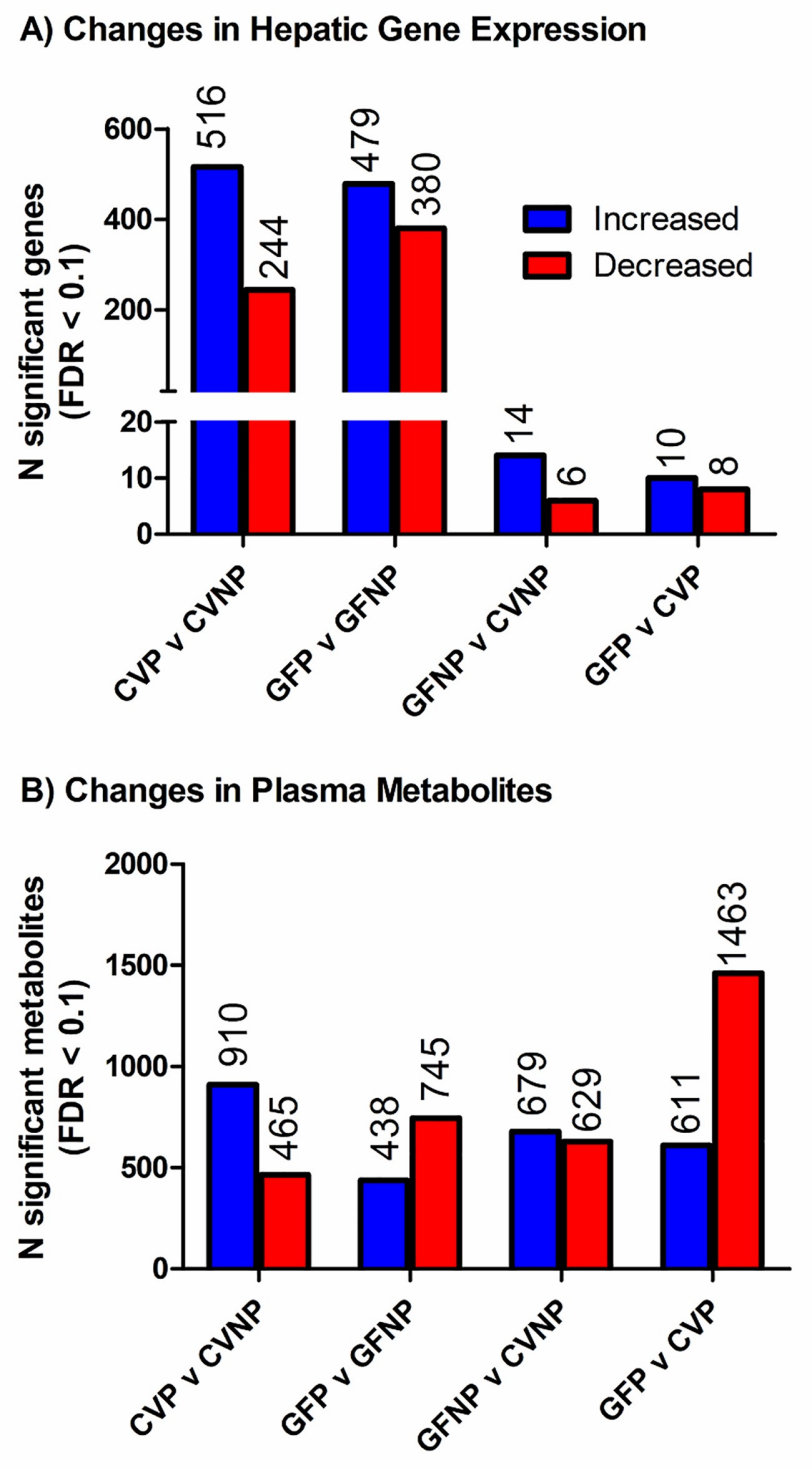
The number of differentially expressed genes and differentially produced metabolites between various comparison mouse groups. Differentially expressed hepatic genes (**A**) and differentially produced metabolites in maternal plasma (**B**) between CVP and CVNP, GFP and GFP, GFNP and CVNP, and GFP and CVP mice. The number of CVNP, CVP, GFNP and GFP mice used was 6, 5, 6, and 5, respectively. Inclusion criteria for genes and metabolites were FDR of 0.1 and a minimum 2-fold change in at least one comparison group.

To identify genes whose liver expression was associated with either pregnancy or the microbiome or both, we performed RNA-seq analysis of liver tissues (n = 6, 5, 6, and 5 for CVNP, CVP, GFNP, and GFP mice, respectively). A total of 1241 genes were significantly changed in at least one comparison group using a threshold of FDR < 0.1 and fold-change > 2. Note that some genes were significantly changed in more than one comparison groups and therefore counted multiple times in Fig 1. Fig 1A illustrates the number of differentially expressed genes (DEGs) when comparing CVP and CVNP, GFP and GFNP, GFNP and CVNP, and GFP and CVP mice. A full list of DEGs is available in S1 Table in our previous study [12]. Of these DEGs, we identified 516 genes that were upregulated and 244 genes that were downregulated by pregnancy in CV mice; whereas 479 genes were upregulated and 380 genes were downregulated by pregnancy in GF mice. We identified 14 upregulated and 6 downregulated genes in GFNP versus CVNP mice, and 10 upregulated and 8 downregulated genes in GFP versus CVP mice. We also examined whether pregnancy-induced changes in hepatic gene expression differ between GF and CV mice by comparing pregnancy-induced DEGs in GF (GFP vs. GFNP) and CV (CVP vs. CVNP) mice, which reflects the interactions between pregnancy and microbiota status (GF or CV). We only detected 3 genes (*Cyp2b13*, *Scd1* and *Lama4*) with FDR < 0.1 and fold-change of >2 for such interactions. Therefore, pregnancy-induced changes in these genes were not included in the pathway analysis (see below).

### Changes in plasma metabolites in CV and GF mice by pregnancy

Next, we identified plasma metabolites associated with pregnancy and/or the microbiome using untargeted LC-MS/MS-based metabolomics. As shown in Fig 1B, we identified a total of 2277 metabolites for which abundances were altered by pregnancy and/or germ-free status, which were considered statistically significant based on FDR < 0.1 and fold-change > 2. A full list of differentially produced metabolites is available in S1 Table. Of these metabolites, there were 910 increased metabolites and 465 decreased metabolites in CVP versus CVNP mice, and 438 increased metabolites and 745 decreased metabolites in GFP versus GFNP mice. In addition, we identified 679 increased metabolites and 629 decreased metabolites in GFNP versus CVNP mice, and 611 increased metabolites and 1463 decreased metabolites in GFP versus CVP mice.

**Fig 2:**
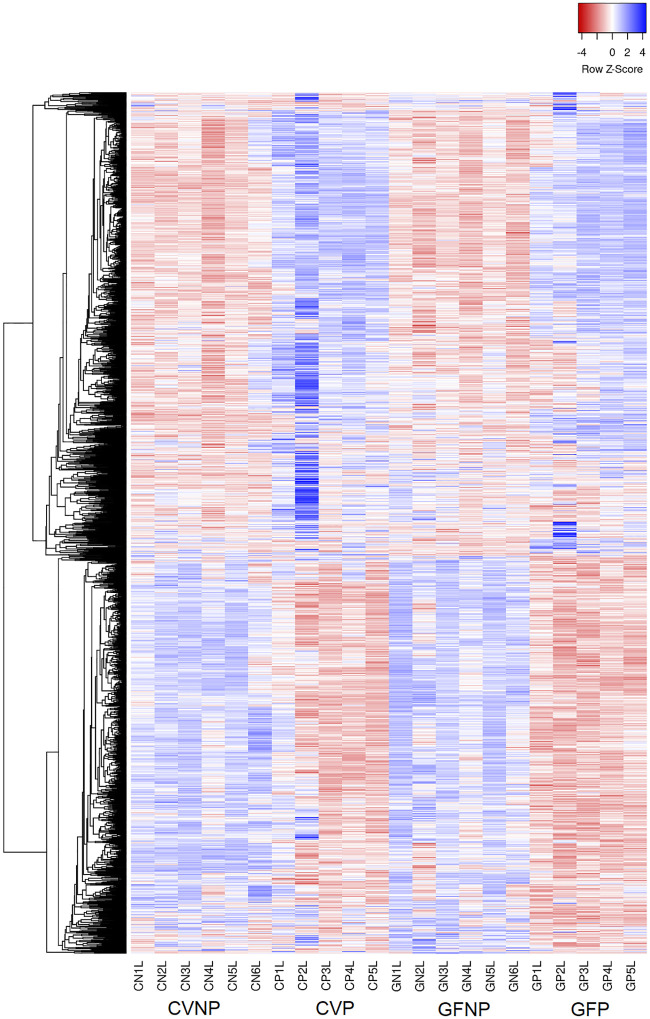
Heatmap of hepatic genes with at least 2-fold change and FDR < 0.1. Inclusion criteria for the genes presented in this heatmap were FDR of 0.1 or less and a minimum 2-fold change in at least one comparison group between CVP and CVNP, GFP and GFP, GFNP and CVNP, and GFP and CVP mice. CVNP, conventional non-pregnant mice; CVP, conventional pregnant mice; GFNP, germ-free non-pregnant mice; GFP, germ-free pregnant mice.

**Fig 3:**
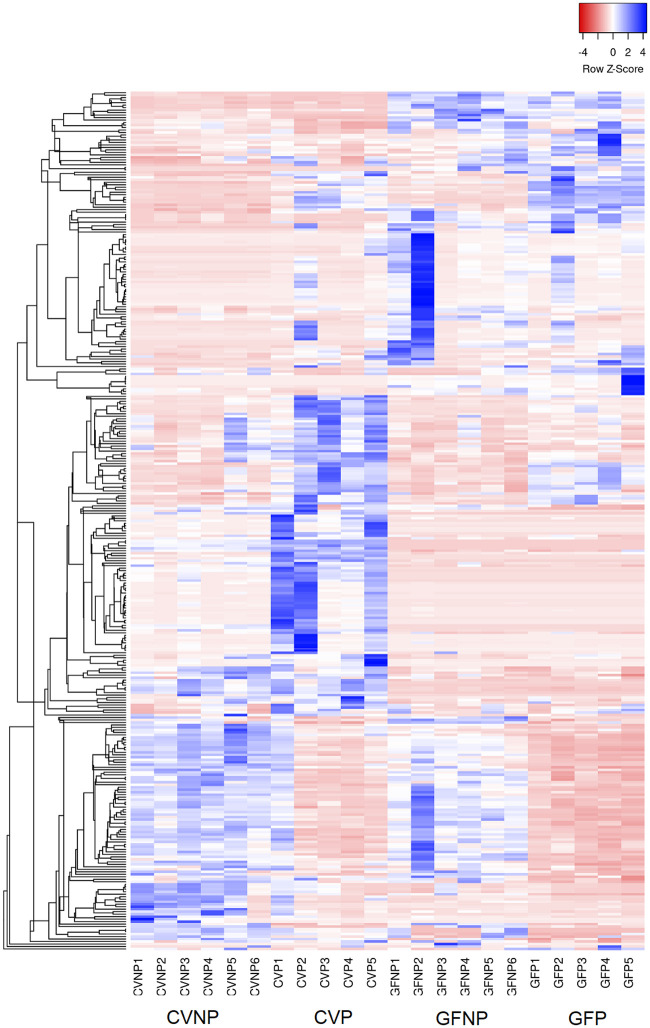
Heatmap of metabolites in maternal plasma with at least 2-fold change and FDR < 0.1. Inclusion criteria for the metabolites presented in this heatmap were FDR of 0.1 of less and a minimum 2-fold change in at least one comparison group between CVP and CVNP, GFP and GFP, GFNP and CVNP, and GFP and CVP mice. CVNP, conventional non-pregnant mice; CVP, conventional pregnant mice; GFNP, germ-free non-pregnant mice; GFP, germ-free pregnant mice.

Taken together, we observed significant associations between pregnancy and the microbiome on both hepatic gene expression and the levels of metabolites in maternal plasma. Whereas hepatic gene expression clusters showed significant differences as a result of pregnancy, the effects of the microbiome were much less pronounced (Fig 2). On the contrary, the metabolite heatmap depicted a number of genes associated with not only pregnancy, but the microbiome (Fig 3).

### Metabolic pathway analysis

**Fig 4:**
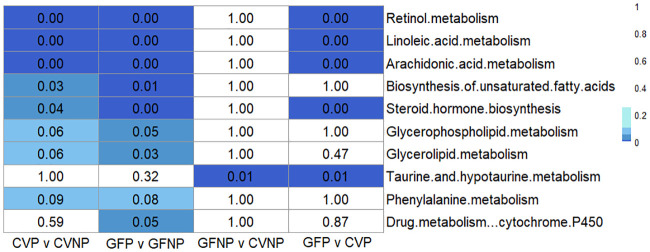
Heatmap of False Discovery Rates (FDRs) of top metabolic pathway hits among all comparison groups. Filtering criterion was 1) genes and metabolites metabolites with FDR of 0.1 of less and a minimum 2-fold change in at least one comparison group between CVP and CVNP, GFP and GFP, GFNP and CVNP, and GFP and CVP mice; and 2) a minimum of 1 gene and 1 metabolite hit per pathway with FDR < 0.1 and a minimum 2-fold change in the comparison groups between CVP and CVNP, GFP and GFP, GFNP and CVNP, and GFP and CVP mice. Those that failed to meet this criterion was labeled as P = 1. CVNP, conventional non-pregnant mice; CVP, conventional pregnant mice; GFNP, germ-free non-pregnant mice; GFP, germ-free pregnant mice.

To identify metabolic pathways altered by pregnancy and/or the microbiome, we performed pathway enrichment analysis that integrated the transcriptomics and metabolomics data using metabo-analyst. All significantly enriched pathways and the number of corresponding gene and metabolites which were associated with each group are summarized in Table 1. All metabolic pathways that are significantly associated with multiple comparisons are summarized in Fig 4. We identified 8 pathways significantly (FDR < 0.1; Fishers Exact Test) enriched for DEGs and metabolites associated with CVP versus CVNP, 9 pathways significantly enriched for DEGs and metabolites associated with GFP versus GFNP, 1 pathway significantly enriched for DEGs and metabolites associated with GFNP versus CVNP, and 5 pathways enriched for DEGs and metabolites associated with GFP versus CVP (Fig 4). Retinol metabolism, linoleic acid metabolism, arachidonic acid metabolism, and steroid hormone biosynthesis were significantly enriched for DEGs and metabolites associated with CVP versus CVNP, GFP versus GFNP, and GFP versus CVP (FDR < 0.1; Fishers Exact Test), but not with GFNP versus CVNP, suggesting that both pregnancy and the microbiome could have a profound impact on these metabolic pathways. The pathways that were significantly enriched for DEGs and metabolites associated with pregnancy in both CV and GF mice (FDR < 0.1; Fishers Exact test), but were not enriched for DEGs and metabolites associated with the germ-free status in pregnant and non-pregnant mice included biosynthesis of unsaturated fatty acids, glycerophospholipid metabolism, glycerolipid metabolism, and phenylalanine metabolism. In contrast, taurine and hypotaurine metabolism were not significantly enriched for DEGs and metabolites associated with pregnancy in both CV and GF mice, but were significantly enriched for DEGs and metabolites associated with the microbiome in pregnant and non-pregnant mice (FDR < 0.1; Fishers Exact Test). The pathway for drug metabolism mediated by cytochrome P450 enzymes was significantly different only between GFP and GFNP mice.

**Table 1:** Top 10 enriched metabolic pathways with the number of corresponding gene and metabolite hits for the comparison groups between CVP and CVNP, GFP and GFNP, GFNP and CVNP, or GFP and CVP mice.

**CVP versus CVNP**					
**Pathway**	**# Gene hits**	**# Metabolite hits**	**p-value**	**FDR**	**Impact**
Retinol metabolism	22	2	2.41E-09	1.90E-07	1.23
Linoleic acid metabolism	9	5	6.22E-08	2.46E-06	2.13
Arachidonic acid metabolism	16	12	5.31E-07	1.40E-05	1.14
Biosynthesis of unsaturated fatty acids	1	9	0.002	0.032	0.46
Steroid hormone biosynthesis	18	20	0.003	0.042	0.76
Glycerophospholipid metabolism	14	8	0.005	0.057	0.86
Glycerolipid metabolism	10	2	0.005	0.057	0.82
Taurine and hypotaurine metabolism	0	3	0.010	0.091	0.40
Phenylalanine metabolism	2	6	0.010	0.091	1.43
Glutathione metabolism	11	0	0.022	0.175	0.24
**GFP versus GFNP**					
**Pathway**	**# Gene hits**	**# Metabolite hits**	**p-value**	**FDR**	**Impact**
Retinol metabolism	23	2	2.17E-14	1.46E-12	1.12
Linoleic acid metabolism	10	5	8.80E-12	2.95E-10	2.13
Arachidonic acid metabolism	11	12	1.22E-06	2.72E-05	0.99
Steroid hormone biosynthesis	17	20	6.37E-06	1.07E-04	0.51
Drug metabolism—other enzymes	14	0	2.03E-04	0.003	0.21
Ascorbate and aldarate metabolism	5	0	6.37E-04	0.007	0.38
Biosynthesis of unsaturated fatty acids	1	9	0.001	0.008	0.67
Glycerolipid metabolism	8	2	0.004	0.032	0.65
Drug metabolism—cytochrome P450	11	1	0.006	0.045	0.18
Glycerophospholipid metabolism	9	8	0.007	0.048	0.71
**GFNP versus CVNP**					
**Pathway**	**# Gene hits**	**# Metabolite hits**	**p-value**	**FDR**	**Impact**
Steroid hormone biosynthesis	0	20	7.62E-09	2.82E-07	0.30
Arachidonic acid metabolism	0	12	5.65E-07	1.01E-05	0.58
Linoleic acid metabolism	0	5	8.17E-07	1.01E-05	1.50
Phenylalanine metabolism	0	6	3.26E-05	3.02E-04	1.09
Biosynthesis of unsaturated fatty acids	0	9	2.11E-04	0.002	0.20
Phenylalanine, tyrosine and tryptophan biosynthesis	0	3	0.001	0.005	1.70
Glycerophospholipid metabolism	0	8	0.001	0.006	0.49
Taurine and hypotaurine metabolism	1	3	0.001	0.006	0.67
Primary bile acid biosynthesis	0	7	0.020	0.083	0.13
Citrate cycle (TCA cycle)	0	4	0.031	0.111	0.56
**GFP versus CVP**					
**Pathway**	**# Gene hits**	**# Metabolite hits**	**p-value**	**FDR**	**Impact**
Arachidonic acid metabolism	4	12	6.75E-10	2.43E-08	0.76
Steroid hormone biosynthesis	4	20	3.30E-09	5.94E-08	0.32
Linoleic acid metabolism	3	5	1.80E-08	2.16E-07	1.75
Retinol metabolism	4	2	2.34E-05	2.11E-04	0.51
Phenylalanine metabolism	0	6	3.26E-05	2.35E-04	1.09
Biosynthesis of unsaturated fatty acids	0	9	2.11E-04	0.001	0.20
Phenylalanine, tyrosine and tryptophan biosynthesis	0	3	0.001	0.005	1.70
Glycerophospholipid metabolism	0	8	0.001	0.005	0.49
Taurine and hypotaurine metabolism	1	3	0.003	0.011	0.67
Primary bile acid biosynthesis	0	7	0.020	0.073	0.13

Inclusion criteria for the gene and metabolite hits were FDR of 0.1 or less and a minimum 2-fold change.

We noted that four metabolic pathways (retinol metabolism, linoleic acid metabolism, arachidonic acid metabolism, and steroid hormone biosynthesis) were not significantly different between GFNP and CVNP mice, but were significantly associated with the pregnancy status in GF mice (GFP versus CVP) (Fig 4), indicating that the effects of the microbiome on these metabolic pathways are unique for pregnancy. Since this finding implies a potential interplay between pregnancy and the microbiome, we elected to perform further in-depth analysis of these pathways for the comparison group of GFP versus CVP mice. Within the linoleic acid metabolism pathway, the plasma levels of 4 metabolites were significantly decreased in GFP versus CVP mice, including linoleate by 63% (FDR < 0.1), phosphatidylcholine by 86% (FDR < 0.1), 9(10)-EpOME by 100% (FDR < 0.1), and 13-Hpode by 66% (FDR < 0.1) (Table 2). Only one metabolite, 12(13)-EpOME, was increased 81-fold (FDR < 0.1) (Table 2). Three genes (*Cyp2c38*, *Cyp2c50 and Cyp2c54*) involved in linoleic acid metabolism were upregulated in GFP versus CVP mice (Table 2). The retinol metabolism pathway was also significantly enriched in GFP compared to CVP mice, with 4 genes upregulated and the levels of 2 metabolites increased in GFP versus CVP mice (Table 2). For arachidonic acid metabolism, the plasma levels of most of the epoxyeicosatrienoic acid (EET) and hydroxyeicosatetraenoic acid (HETE) metabolites were prominently decreased in GFP versus CVP mice, with the exception of arachidonate that was increased 3.1-fold (FDR < 0.1). Within the steroid hormone biosynthesis pathway, there were 20 differentially abundant metabolites, with certain metabolites increased and some metabolites decreased in GFP versus CVP mice. Notably, the plasma levels of corticosterone, cortisol, and their subsequent metabolites were increased 1.2–4.2-fold (FDR < 0.1). In contrast, allopregnanolone exhibited a 100% decrease in GFP versus CVP (FDR < 0.1). Interestingly, the same genes (*Cyp2b13*, *Cyp2c38*, *Cyp2c50* and *Cyp2c54* for retinol metabolism, arachidonic acid metabolism, and steroid hormone biosynthesis, as well as *Cyp2c38*, *Cyp2c50* and *Cyp2c54* for linoleic acid metabolism) were upregulated 2.0–5.3-fold (FDR < 0.1) in all the significantly enriched pathways (Table 2). The full pathways of linoleic acid metabolism, retinol metabolism, arachidonic acid metabolism, and steroid hormone biosynthesis are shown in S1–S4 Figs, respectively.

**Table 2:** Significantly changed metabolic pathways with gene and metabolite hits in GFP mice versus CVP mice.

Pathway	FDR	Impact	Gene Hits		Metabolite Hits	
			Gene	Fold Change	Metabolite	Fold Change
Retinol metabolism	2.11E-04	0.51	Cyp2b13	5.28	Retinoate	3.36
			Cyp2c38	3.03	9-cis-Retinoic acid	3.36
(mmu00830)			Cyp2c50	2.03		
			Cyp2c54	2.22		
Linoleic acid metabolism	2.16E-07	1.75	Cyp2c38	3.03	Linoleate	0.37
			Cyp2c50	2.03	Phosphatidylcholine	0.14
(mmu00591)			Cyp2c54	2.22	9(10)-EpOME	0.00
					12(13)-EpOME	81.0
					13-Hpode	0.34
Arachidonic acid metabolism	2.43E-08	0.76	Cyp2b13	5.28	5,6-EET	0.48
			Cyp2c38	3.03	8,9-EET	0.48
			Cyp2c50	2.03	11,12-EET	0.48
(mmu00590)			Cyp2c54	2.22	14,15-EET	0.48
					Arachidonate	3.07
					Phosphatidylcholine	0.14
					Leukotriene A4	0.84
					16(R)-HETE	0.48
					20-HETE	0.48
					15(S)-HETE	0.48
					19(S)-HETE	0.48
					5(S)-HETE	0.48
Steroid hormone biosynthesis	5.94E-08	0.32	Cyp2b13	5.28	11β,17α,21-Trihydroxypregnenolone	0.84
			Cyp2c38	3.03	16α-Hydroxydehydroepiandrosterone	0.84
			Cyp2c50	2.03	Corticosterone	4.23
(mmu00140)			Cyp2c54	2.22	Aldosterone	1.73
					11β-Hydroxyprogesterone	1.97
					Allopregnanolone	0.00
					Cortisol	1.19
					11-Deoxycortisol	4.23
					Cortisone	1.73
					21-Deoxycortisol	4.23
					2-Methoxyestrone	1.73
					18-Hydroxycorticosterone	2.41
					19-Oxoandrost-4-ene-3,17-dione	1.73
					19-Hydroxytestosterone	0.84
					11β,21-Dihydroxy-3,20-oxo-5β-pregnan-18-al	0.84
					11-Dehydrocorticosterone	2.41
					Dihydrocortisol	0.84
					17α,21-Dihydroxy-5β-pregnane-3,11,20-trione	2.41
					Adrenosterone	1.73
					7α-Hydroxydehydroepiandrosterone	0.84

Corresponding gene and metabolite hits that were differentially changed in each pathway are detailed with fold changes. Impact score was calculated based on degree centrality algorithms, and FDR values were determined based on pathway-level weighting. Inclusion criteria for the gene and metabolite hits presented in this table were FDR of 0.1 or less and a minimum 2-fold change in at least 1 mouse group comparison.

## Discussion

Pregnancy imposes substantial adaptive metabolic changes to the mother to maintain the wellbeing of herself and her fetus [19, 20]. Recent studies have extensively discussed the potential for the gut microbiome to modulate host metabolism of endogenous and exogenous substances [5]. Very little is known about how the microbiome alters host metabolic processes during pregnancy. Therefore, in this study, we explored changes within metabolic pathways related to the microbiome in pregnancy using CV and GF pregnant mouse models.

We used an approach that integrated changes in both hepatic gene expression and maternal plasma metabolites. Overall, we observed similar changes in metabolic pathways associated with pregnancy in CV and GF mice, with 8 metabolic pathways for endogenous compounds enriched for DEGs associated with the pregnancy status in both groups, and only 1 pathway (Drug metabolism by cytochrome P450) was uniquely enriched for DEGs associated with pregnancy in GF mice (Fig 4 and Table 1). The 8 metabolic pathways that were significantly enriched include retinol metabolism, linoleic acid metabolism, arachidonic acid metabolism, biosynthesis of unsaturated fatty acids, steroid hormone biosynthesis, glycerophospholipid metabolism, glycerolipid metabolism, and phenylalanine metabolism. Of the 8 metabolic pathways, the changes in retinol metabolism by pregnancy were most notable in both CV and GF mice. Retinol, also known as vitamin A, is believed to be critical for healthy fetal development [21–23]. Likewise, linoleic acid metabolism, arachidonic acid metabolism, and biosynthesis of unsaturated fatty acids were also significantly altered by pregnancy in both CV and GF mice. All three metabolic pathways are essential for providing energy and nutrition to support intrauterine growth [24]. Steroid hormone biosynthesis which was also enriched for DEGs associated with pregnancy in both CV and GF mice is also known to be essential for maintaining healthy pregnancy, from before the point of conception, during fertilization, and throughout gestation [25]. As all these metabolic pathways are essential for a successful pregnancy and fetal development, it is not surprising that we observed significant changes in these pathways by pregnancy regardless of the germ-free status. Changes in these pathways by pregnancy reflect metabolic response of the maternal body to the rapidly growing fetus and its nutritional demands. We recognize that the gestation day variability due to difficulties achieving pregnancy in GF mice is a limitation of this study. While the gestation day of CV mice was gd 15, the range of gestation day of GF mice in this study was between gd 14–16. In a previous study, we found that between gd 13–19, the expression levels of the majority metabolic enzyme and transporter genes in the liver remained relatively stable with no more than a 2-fold difference [26]. Furthermore, this study showed that there were no major differences with respect to the top eight metabolic pathways shown in Fig 4 that were enriched by pregnancy between CV and GF mice. Based on these data, the gestation day variation in GF mice appears to have a minor impact on the results obtained regarding the metabolic pathways important for pregnancy and fetal growth.

We further analyzed the impact of microbiome on metabolic pathways in pregnant mice by comparing metabolic pathways in GFP mice versus CVP mice. Among the 8 pathways significantly enriched for DEGs and metabolites associated with pregnancy regardless in CV and GF mice, 4 pathways, including retinol metabolism, linoleic acid metabolism, arachidonic acid metabolism, and steroid hormone biosynthesis, were also enriched for DEGs and metabolites associated with the germ-free status in pregnant mice (Fig 4). Notably, these 4 metabolic pathways were not enriched for DEGs and metabolites with the germ-free status at all in non-pregnant mice (Fig 4), suggesting that the effects of the lack of microbiome on these pathways are unique for pregnancy. This could be due to the shift in the microbiome composition by pregnancy as previously reported [10]. As mentioned above, all the 4 metabolic pathways are important for successful pregnancy and fetal development. Since our study endpoints did not include health outcomes of the pregnancy, it is unclear what impact the microbiome-mediated changes in these metabolic pathways would have on the overall maternal and fetal health. In this regard, we noted that GF C57BL/6 mice used in this study had a much lower breeding ability compared to CV C57BL/6J mice. Thus, it is important to further investigate in future studies as to whether and how the pregnancy-specific effects of the microbiome on host hepatic metabolism impact maternal health and fetal development, including the reduced breeding ability.

To dissect the results more, we identified all plasma metabolites that were affected in each of the four metabolic pathways. Phosphatidylcholine was mapped to both linoleic acid metabolism and arachidonic acid metabolism pathways and was markedly decreased by 86% in GFP versus CVP mice (Table 2). Phosphatidylcholine is a major component of the phospholipid membrane of eukaryote cells and has been speculated to be an important mediator of the symbiotic relationship between bacteria and host [27]. While phosphatidylcholine is usually obtained via foods such as eggs and soybeans in humans, bacteria are also capable of the biosynthesis of phosphatidylcholine via phospholipid *N*-methylation pathway and the CDP-choline pathway [27–29]. Thus, a marked decrease in phosphatidylcholine would be expected in GFP mice. Another significantly decreased (by 100%) metabolite in GFP versus CVP mice was allopregnanolone, a component of the steroid hormone biosynthesis pathway. Previous studies have suggested that allopregnanolone is a ligand that can potentially activate the nuclear receptor PXR at micromolar concentrations [30]. A significant decrease in this PXR ligand may explain a decrease in gene expression of some PXR-activated drug processing genes such as *Cyp3a11* [12]. It is interesting and novel that this study showed that several steroids belonging to glucocorticoids including corticosterone, 11-deoxycortisol, 21-deoxycortisol, 18-hydroxycorticosterone, and 11-dehydrocorticosterone were increased 2-5-fold in GFP mice versus CVP mice (Table 2). Production of physiologically active glucocorticoids such as corticosterone is increased during pregnancy, which is essential for fetal development [31, 32]. The impact of increased production of glucocorticoids as a result of the lack of microbiome during pregnancy on maternal and fetal physiology remains to be determined. We identified a dramatic 81-fold increase of 12(13)-EpOME (the 12,13-cis epoxide form of linoleic acid) in GFP mice versus CVP mice (Table 2). 12(13)]-EpOME is produced by neutrophils during respiratory burst [33]. Elevated plasma EpOME levels are associated with acute respiratory distress syndrome, a systemic failure of organ systems frequently observed in trauma victims [34]. This drastic increase in 12(13)-EpOME is striking, and could be an indicator of an exacerbated immune response or inflammation in GF mice during pregnancy. We recognize that the untargeted metabolomics analysis of this study revealed relative changes, and therefore the data obtained for certain metabolites would require validation by absolute quantification of the metabolites, which is an important topic of future studies. Nevertheless, the trend in changes of many metabolites by pregnancy such as glucocorticoids is consistent with literature data. Taken together, the results of this study suggest that the microbiome may have a significant impact on endogenous metabolic processes that are critical for a healthy pregnancy and fetal development.

Intriguingly, we found that the same genes, *Cyp2b13*, *Cyp2c38*, *Cyp2c50*, and *Cyp2c54*, in the 4 metabolic pathways were all significantly induced in GFP versus CVP mice (Table 2). Of the four genes, only *Cyp2c50* is a known to have a clear human homolog, CYP2C19 [35]. CYP2C19 activity in humans is known to decrease during pregnancy [36]. Our previous study also showed downregulation of *Cyp2c50* in pregnancy, regardless of the microbiome status [12]. Cyp2c50 plays an important role as arachidonic acid epoxygenase and is considered a major metabolizing enzyme for the production of epoxyeicosatrienoic acids (EETs) [37]. We observed an overall decrease in EETs in GFP compared to CVP mice, which is opposite to what we would expect due to induction of *Cyp2c50*. The increase in arachidonate in GFP vs. CVP mice is likely the consequence of the overall decrease in EETs (metabolites of arachidonate) in GFP vs. CVP mice. *Cyp2c50* is also known to mediate linoleic acid metabolism [38]. We observed that the downstream metabolite, 9(10)-EpOME, was significantly decreased in GFP compared to CVP mice (Table 2), which is also opposite to the induction of *Cyp2c50*. However, induction of *Cyp2c50* may contribute to the drastic increase in 12(13)-EpOME in GFP vs. CVP. Overall, these data on plasma metabolites seem to suggest altered (lower or higher) enzymatic activity of CYP2C50 in GFP vs. CVP mice, yet its mRNA expression was induced in GFP vs. CVP mice. Such a disconnection between mRNA expression of gene and its metabolites warrants further investigation in future studies.

In summary, to our knowledge, this is the first study to provide novel information on the interplay between the microbiome and pregnancy to affect multiple hepatic metabolic pathways in mouse models. The pathways uniquely affected by the microbiome during pregnancy include retinol metabolism, linoleic acid metabolism, arachidonic acid metabolism, and steroid hormone biosynthesis, which are all critical for normal pregnancy and fetal development, suggesting that the microbiome may play an important role in maternal and fetal health. While these results provide novel insight into the role of the microbiome in modulating host metabolic processes during pregnancy, caution should be taken for interpretation and extrapolation of the mouse data to humans for several reasons. First, this study used a complete germ-free mouse model. We cannot differentiate between the effects of the gut versus other microbiota in the dams (e.g., vaginal) that may also have contributed to the effects of microbiome we observed. Indeed, cervicovaginal microbiome composition has been shown to be associated with metabolic profiles in healthy pregnancy [39]. Also, the lack of microbiota from birth in the germ-free mouse model may influence the proper development of immune system, thus affecting healthy pregnancy. Second, there have been reports of decrease in energy absorption from diet in GF mice as a result of the lack of microbiome, which may contribute to potential differences in energy pathway modulation between CV and GF mice [40]. However, we did not observe significant impacts of the microbiome on these pathways. Third, the human microbiome composition is vastly different from that of mice, and a germ-free status can never be achieved in pregnant women. Forth, the choice of only one gestation day (gd 15) for tissue sampling in this study may not have captured all the dynamic changes that would occur throughout gestation. Future studies should be done with multiple gestational time points to help construct a more dynamic profile of microbiome-mediated changes in metabolic pathways throughout gestation. Finally, given the vast differences in the gut microbiome composition between different mouse models [41], and that diet is a potential source of external factor that could lead to differences in the gut microbiome composition in mice [42], the findings of this study could possibly be dependent on mouse breeds and/or diet as well. The current study serves as a proof-of-concept for future studies, and the results obtained in this study establish the basis for further investigation of the role of the microbiome in altering host metabolism during pregnancy and for elucidating the molecular mechanisms behind these metabolic changes.

## Supporting information

S1 Fig Linoleic acid metabolism KEGG pathway. Hepatic genes are illustrated in green boxes and metabolites are presented as circles. Orange highlights are those enriched by analysis.
(PDF)

S2 Fig Retinol metabolism KEGG pathway. Hepatic genes are illustrated in green boxes and metabolites are presented as circles. Orange highlights are those enriched by analysis.
(PDF)

S3 Fig Arachidonic acid metabolism KEGG pathway. Hepatic genes are illustrated in green boxes and metabolites are presented as circles. Orange highlights are those enriched by analysis.
(PDF)

S4 Fig Steroid hormone biosynthesis KEGG pathway. Hepatic genes are illustrated in green boxes and metabolites are presented as circles.
(PDF)

S1 Table A full list of differentially produced metabolites. (XLSX)
