# Supplementary material for: Key hepatic metabolic pathways are altered in germ-free mice during pregnancy
Source: PLoS One. 2021 Mar 12;16(3):e0248351. doi: 10.1371/journal.pone.0248351 (PMC7954286; doi:10.1371/journal.pone.0248351)
Supplement: S1 Fig — Hepatic genes are illustrated in green boxes and metabolites are presented as circles. Orange highlights are those enriched by analysis. (PDF) [file pone.0248351.s001.pdf]

S1 Figure. Linoleic acid metabolism

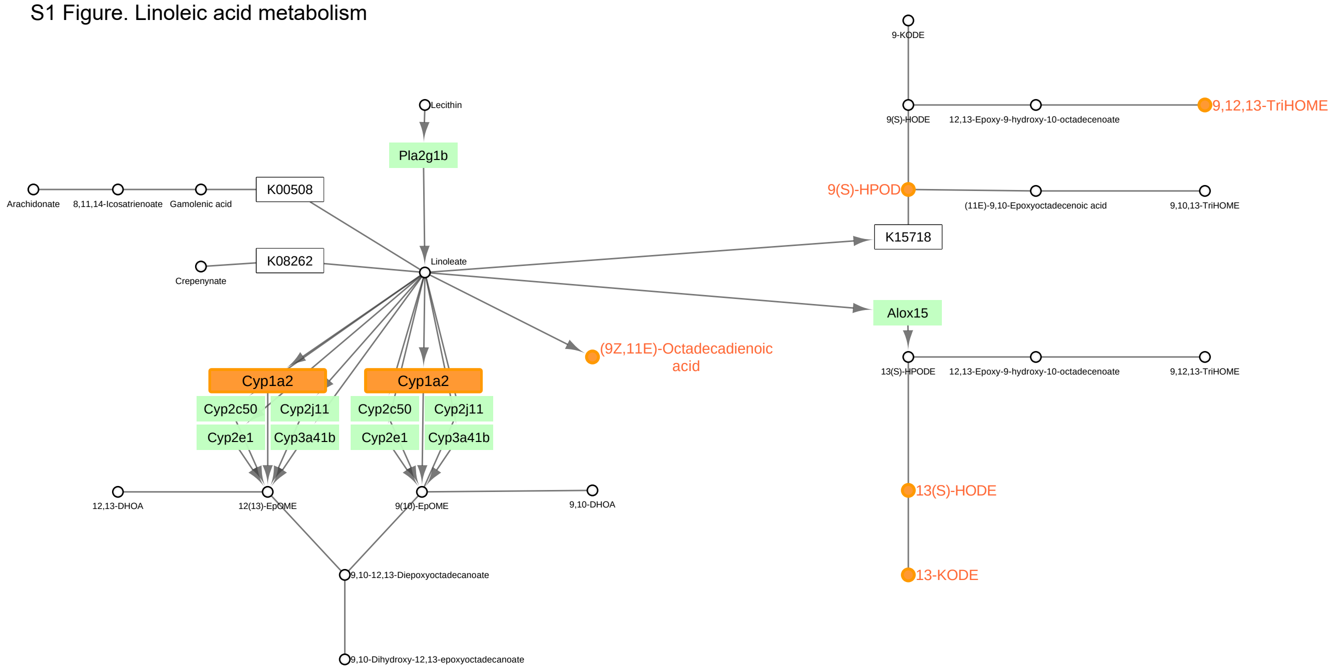

S1 Figure. Linoleic acid metabolism KEGG pathway. Hepatic genes are illustrated in green boxes and metabolites are presented as circles. Orange highlights are those enriched by analysis.
