# Supplementary material for: Key hepatic metabolic pathways are altered in germ-free mice during pregnancy
Source: PLoS One. 2021 Mar 12;16(3):e0248351. doi: 10.1371/journal.pone.0248351 (PMC7954286; doi:10.1371/journal.pone.0248351)
Supplement: S3 Fig — Hepatic genes are illustrated in green boxes and metabolites are presented as circles. Orange highlights are those enriched by analysis. (PDF) [file pone.0248351.s003.pdf]

S3 Figure Arachidonic Acid metabolism

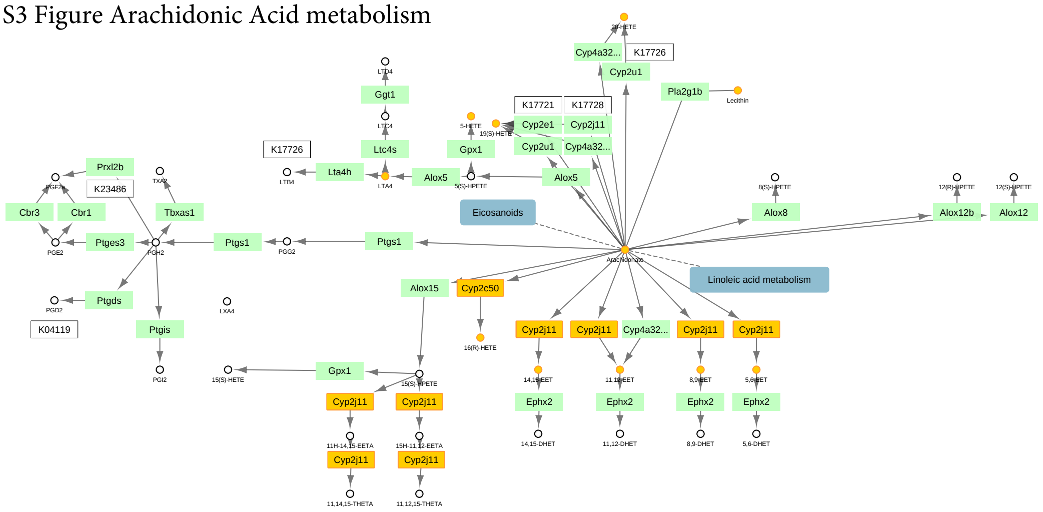


S3 Figure. Arachidonic acid metabolism KEGG pathway. Hepatic genes are illustrated in green boxes and metabolites are presented as circles. Orange highlights are those enriched by analysis.
