# Supplementary material for: Key hepatic metabolic pathways are altered in germ-free mice during pregnancy
Source: PLoS One. 2021 Mar 12;16(3):e0248351. doi: 10.1371/journal.pone.0248351 (PMC7954286; doi:10.1371/journal.pone.0248351)
Supplement: S4 Fig — Hepatic genes are illustrated in green boxes and metabolites are presented as circles. (PDF) [file pone.0248351.s004.pdf]

S4 Steroid Hormone Biosynthesis

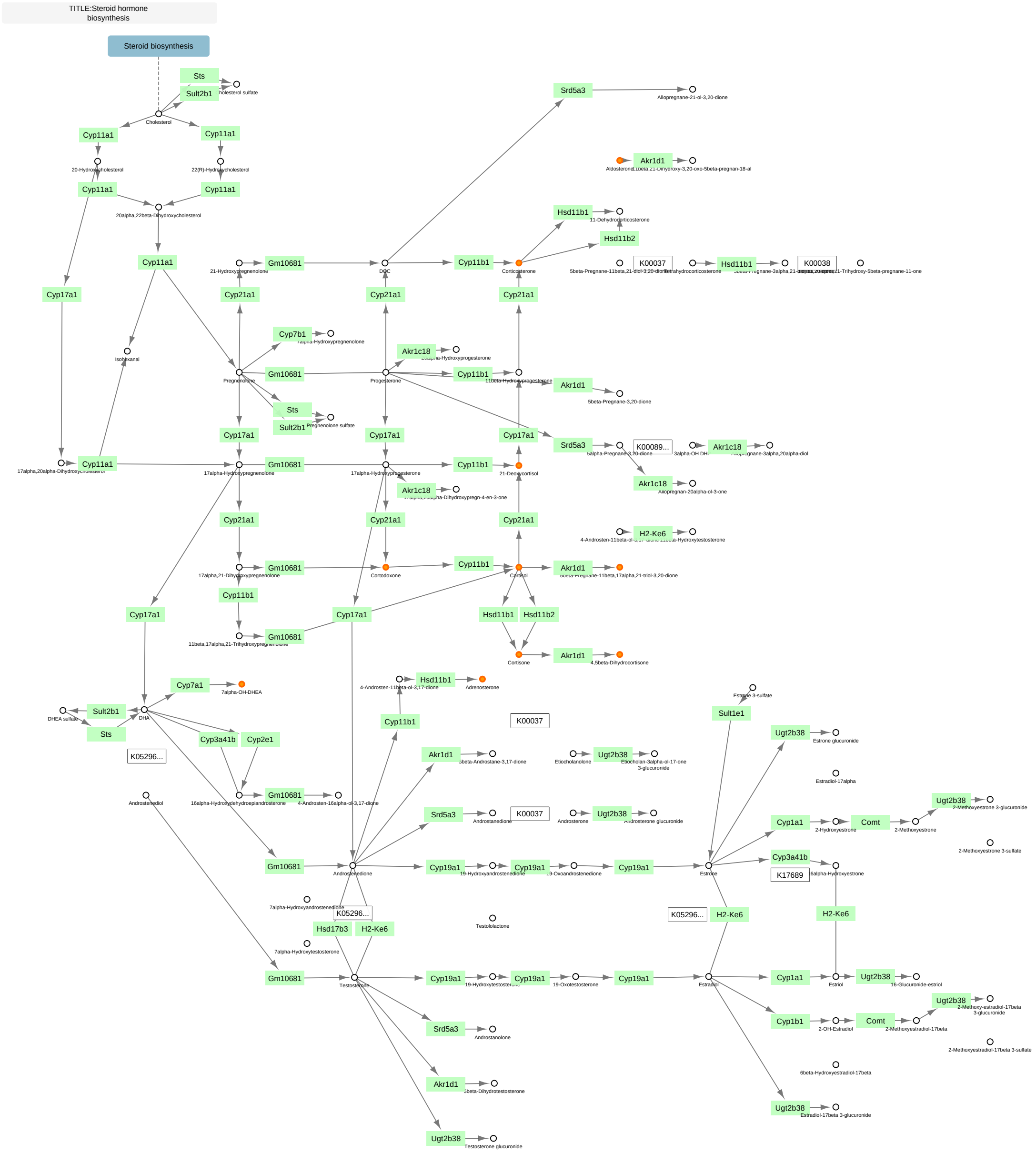

S4 Figure. Steroid hormone biosynthesis KEGG pathway. Hepatic genes are illustrated in green boxes and metabolites are presented as circles.
